# Impacts of Different Prenatal Supplementation Strategies on the Plasma Metabolome of Bulls in the Rearing and Finishing Phase

**DOI:** 10.3390/metabo13020259

**Published:** 2023-02-10

**Authors:** Guilherme Henrique Gebim Polizel, Arícia Christofaro Fernandes, Édison Furlan, Barbara Carolina Teixeira Prati, José Bento Sterman Ferraz, Miguel Henrique de Almeida Santana

**Affiliations:** 1 Department of Animal Science, Faculty of Animal Science and Food Engineering—USP, Av. Duque de Caxias Norte, 225, Pirassununga 13635-900, SP, Brazil; 2 Department of Basic Sciences, Faculty of Animal Science and Food Engineering—USP, Av. Duque de Caxias Norte, 225, Pirassununga 13635-900, SP, Brazil

**Keywords:** beef cattle, fetal programming, metabolism, metabolomics, nutrigenomics

## Abstract

This study investigated the effects of maternal nutrition on the plasma metabolome of Nellore bulls in the rearing and finishing phases, and metabolic differences between these phases. For this study, three nutritional approaches were used in 126 cows during pregnancy: NP—(control) mineral supplementation; PP—protein-energy supplementation in the final third; and FP—protein-energy supplementation during the entire pregnancy. We collected blood samples from male offspring in the rearing (450 ± 28 days old) and finishing phases (660 ± 28 days old). The blood was processed, and from plasma samples, we performed the targeted metabolome analysis (AbsoluteIDQ^®^ p180 Kit). Multiple linear regression, principal component analysis (PCA), repeated measures analysis over time, and an enrichment analysis were performed. PCA showed an overlap of treatments and time clusters in the analyses. We identified significant metabolites among the treatments (rearing phase = six metabolites; finishing phase = three metabolites) and over time (21 metabolites). No significant metabolic pathways were found in the finishing phase, however, we found significant pathways in the rearing phase (Arginine biosynthesis and Histidine metabolism). Thus, prenatal nutrition impacted on plasma metabolome of bulls during the rearing and finishing phase and the different production stages showed an effect on the metabolic levels of bulls.

## 1. Introduction

The main economic interest traits in beef cattle production (growth and muscle development) are dependent on genetics and post-natal environmental conditions [1,2]. However, prenatal life can also impact these characteristics and others as immunology, stress response, reproduction, microbiota, organ development, and metabolic changes [3,4,5,6,7,8,9,10,11,12]. These alterations may persist long-term in an offspring’s life—a concept known as fetal programming.

In beef cattle production, fetal programming may affect several phenotypes [13,14,15,16]. Nevertheless, the mechanisms that involve the responses to each prenatal environment, breed, and other variables have not been elucidated yet. Studies seeking molecular perspectives on phenotypes of interest in beef cattle, such as feed efficiency [17] and meat quality [18], have been increasing together with the decrease in the costs of omics technologies. The omics sciences refer to the study of physiological functions, biological processes, systems, and molecular structures [19]. Omics technologies primarily encompass genomics, transcriptomics, proteomics, and metabolomics [20,21]. The methods used by omics technologies (high-throughput technologies, next-generation sequencing, gas chromatography, liquid chromatography, mass spectrometry, etc.) are able to explore the genome, transcriptome, proteome, and metabolome more widely and efficiently, identifying biomarkers and making molecular diagnoses precisely [22].

The metabolome is the complete collection of metabolites present in a given tissue, cell, organ, or biofluid [23]. This omics science can investigate in depth the effects of prenatal nutrition on the progeny [24], and can significantly contribute to elucidating the metabolic mechanisms involved in the biological responses of the progeny to the nutritional stimulus. However, data from metabolomics are complex (systemic interactions, missing values, noise, etc.) and continuous improvements in pipelines are necessary to evaluate the information that will be generated. Through “machine learning” techniques (supervised and unsupervised), metabolomics data can have greater support for understanding the interactions of this dataset [25].

Thus, we hypothesized that different prenatal supplementation strategies influence the plasma metabolome of Nellore bulls in the rearing and finishing phase. Our objective was to evaluate the long-term effect of prenatal nutrition on plasma metabolome in each phase (rearing and finishing) and assess the metabolic differences over time through supervised and unsupervised machine learning techniques.

## 2. Material and Methods

### 2.1. Experimental Design

A herd of 126 Nellore cows was artificially inseminated using semen from four Nellore bulls. After pregnancy diagnosis, the cows were divided into three treatments (NP—Not programmed, PP—Partial Programming and FP—Full Programmed) based on age, body weight (BW) and body condition score. NP (control) cows received only mineral supplements throughout their pregnancy period (0.03% of BW per day). The PP treatment received protein-energy supplementation (0.3% of BW per day) only in the third trimester of pregnancy, while the FP group received this supplementation (0.3% of BW per day) from the confirmation of pregnancy (30 days) until calving. The three groups received mineral supplementation (0.03% of BW; already included in the protein-energy supplement; Table 1) for the entire period, a practice commonly performed in Brazil due to mineral deficiencies in tropical pastures, particularly during winter.

More information about the phenotypic effects of treatments on dams and details about the paddocks (*Brachiaria brizantha* cv. Marandu) can be found in Schalch Junior et al. [7].

After calving, dams and calves (both males and females) were kept together, despite the nutritional plan, until weaning at 240 ± 28 days old. During the calving to weaning period, all the cows received the same nutritional protocol (mineral supplementation of 0.03% of BW) as during the pregnancy period. After weaning, the calves were divided by sex (males and females), regardless of the treatment, and remained until the end of the rearing phase at 570 ± 28 days old. From calving, the male progeny was submitted to the same environmental conditions (sanitary and nutritional). During the rearing period, the young bulls received two types of supplements: an energetic supplement in the dry season (winter); and a protein supplement in the wet season (summer). Details of both supplements can be found in Polizel et al. [8]. From calving to the start of the finishing phase, the young bulls grazed on *Brachiaria brizantha* cv. Marandu pastures with water *ad libitum.*

The 63 bulls started the finishing phase (in feedlot paddocks) at 570 ± 28 days old and were slaughtered at 676 ± 28 days old by a pneumatic stunner. During this period, three distinct diets were given to the bulls: an adaptation diet (diet 1) for the first 15 days; a second one for the following 35 days; and a third one for the final 56 days. Once the finishing phase was complete, the animals were slaughtered at the FZEA/USP school slaughterhouse. The procedures (slaughter and processing of the carcasses) were performed according to the regulations established by the Ministry of Agriculture, Livestock and Supply of Brazil (MAPA, Normative Instruction No. 9 of 2004).

More details about the phenotypic effects on the bulls, and the finishing and rearing phase can be found in Polizel et al. [8,9,10].

### 2.2. Plasma Sample Collection and Processing

At 450 ± 28 days old and 660 ± 28 days old, the blood samples of the 63 bulls were collected. From this, 5 samples were randomly selected per treatment (*n* = 15) in the rearing phase, and another 5 samples from the same experimental units were selected in the finishing phase (*n* = 15) for carrying out this study. Blood was collected from the jugular vein in EDTA-coated tubes (BD Vacutainer, São Paulo, Brazil) and stored on ice until processing in the laboratory. The samples were centrifuged at 3000× *g* and 4 °C for 10 min within an hour of collection. The plasma supernatants were then transferred to fresh collection tubes and immediately frozen with dry ice before being stored at −80 °C until use.

### 2.3. Targeted Metabolomics

The AbsoluteIDQ^®^ p180 Kit (Biocrates Life Sciences, Innsbruck, Austria) was employed for targeted metabolomics analysis of the plasma samples. This kit quantifies 188 metabolites, including 21 amino acids, 21 biogenic amines, 40 acylcarnitines (Cx:y), 14 lysophosphatidylcholines (lysoPC), 76 phosphatidylcholines (PC), and 15 sphingolipids (SMx:y). The analysis was conducted by Apex Science (Campinas, São Paulo, Brazil). The kit is a combined flow injection (FIA) and liquid chromatography (LC) tandem mass spectrometry assay. The amino acids and biogenic amines were analyzed by liquid chromatography tandem-mass spectrometry (HPLC-MS/MS) with electrospray ionization. The lysophosphatidylcholines, phosphatidylcholines, acylcarnitines, and hexose were evaluated by flow injection analysis-tandem mass spectrometry (FIA-MS/MS). Internal standards, analyte derivatization, and metabolite extraction are integrated into a 96-well plate kit. Mass detection and compound identification were performed by multiple reaction monitoring. Briefly, after the addition of 10 μL of supplied internal standard solution to each well on the filter spot of the 96-well extraction plate, 10 μL of each plasma sample, quality control (QC) samples, or calibration standard was added to the appropriate wells. The plate was dried under a gentle stream of nitrogen. Then, amino acid and biogenic amines were derivatized with phenyl isothiocyanate (Sigma Aldrich, Germany), and dried again. Metabolite extraction was performed with 5 mM ammonium acetate in methanol. The final extracts were analyzed after appropriate dilution by HPLC-MS/MS (amino acids and biogenic amines) and FIA-MS/MS (lysophosphatidylcholines, phosphatidylcholines, acylcarnitines, and hexose). The software MetIDQ^®^ v1.0 performed the metabolite quantification and quality assessment. Biocrates experimentally determines the metabolite-specific limits of detection (LOD) of the assay.

### 2.4. Statistical Analysis

Data processing and the univariate analysis (supervised technique; multiple linear regression) were performed using the “LM” function in the R software environment (version 4.1.2) (https://www.r-project.org/ accessed on 1 January 2023). Metabolites with more than 70% of samples below LOD were removed (filtering data) from the dataset (Rearing phase = 168 metabolites remaining; and Finishing phase = 171 metabolites remaining). The LOD values that remained in the dataset after filtering were replaced by the mean of each variable.

The statistical model used in both production phases (rearing and finishing phase) was:

(1) $$Y_{\mathrm{jk}}=\;\mu +{\;\beta }_{1}{\mathrm{Age}}_{b1}+{\;\mathrm{Treat}}_{j}+e_{\mathrm{jk}}$$

where: Y_jk_ are the observed metabolite from k^th^ animal, recorded on j^th^ treatment; μ is a constant; β_1_ is the regression coefficient of covariate animal’s age; Age_b1_ is the observed value for bull’s age of k^th^ animal; Treat_j_ is the fixed effect of j^th^ treatment; and e_jk_ is the residual random term. The residuals were tested for homoscedasticity (Levene’s test) and for normality (Shapiro-Wilk test), and the differences between treatments were considered significant when *p* ≤ 0.05 by the Tukey Kramer test.

In addition, the metabolite concentration table was uploaded to MetaboAnalyst 5.0 [26], and the data were Auto-scaled (mean-centered and divided by the standard deviation of each variable) before analysis. We performed a principal component analysis (PCA; unsupervised method) of each phase and between both (rearing and finishing phase), an enrichment analysis and a repeated measures analysis over time. The PCA was performed to assess the clusters between treatments (NP, PP, and FP) in each production phase and to evaluate the differences between these stages. The enrichment analysis was carried out by MetaboAnalyst to identify the most relevant biological processes associated with the differentially expressed metabolites (identified in univariate analysis) based on the Kyoto Encyclopedia of Genes and Genomes database (KEGG Pathway). Biological processes with *p* ≤ 0.05 were considered significant. The repeated measures analysis over time was performed by the linear model with the covariate adjustment function of MetaboAnalyst. This analysis considered variables in the model just the time, treatment, and the interaction between both. This approach allows using linear models (limma or lm) to perform significance testing with covariate adjustment.

## 3. Results

### 3.1. Unsupervised Analysis of Metabolome (PCA)

**Figure 1:**
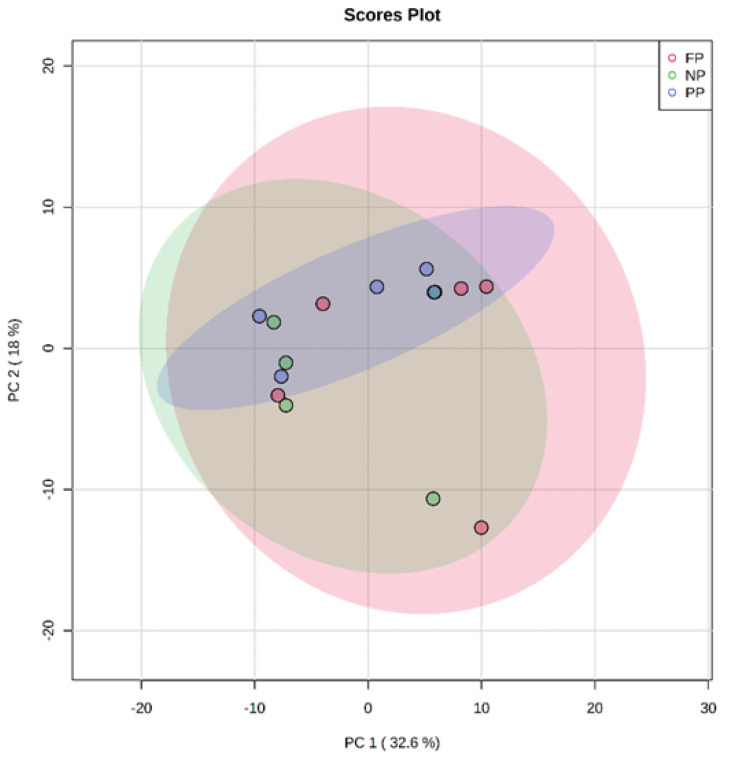
Principal component analysis (PCA) scores plot of metabolome distribution of bulls’ blood plasma in the rearing phase among the treatments (NP, PP, and FP).

**Figure 2:**
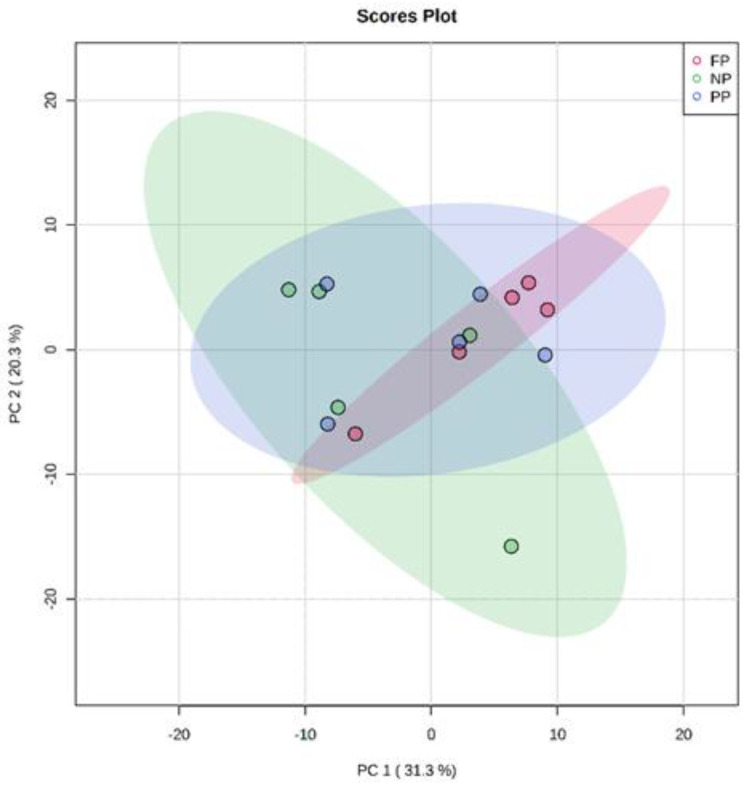
Principal component analysis (PCA) scores plot of metabolome distribution of bulls’ blood plasma in the finishing phase among the treatments (NP, PP and FP).

**Figure 3:**
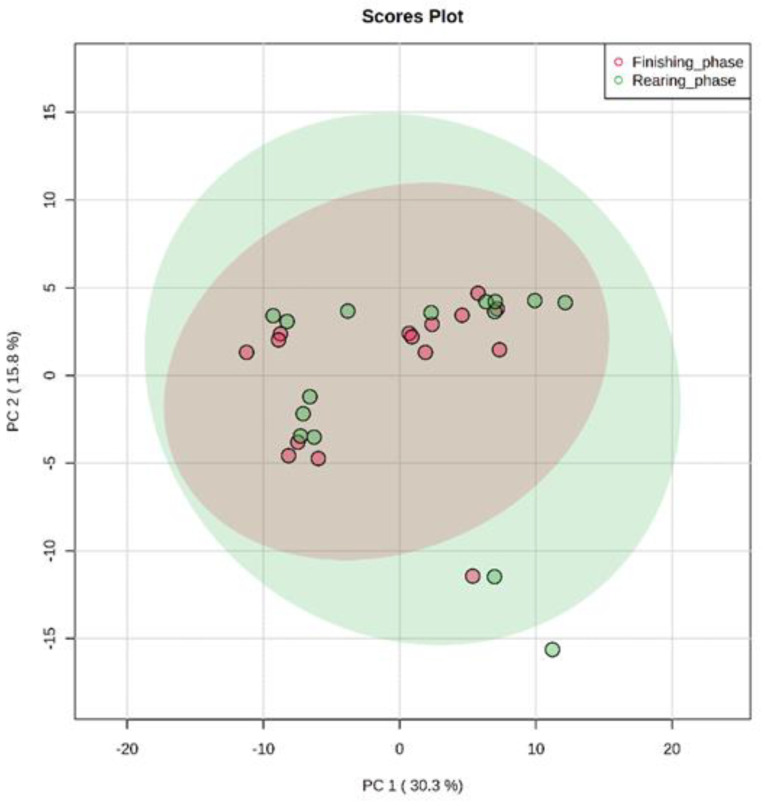
Principal component analysis (PCA) scores plot of metabolome distribution of bulls’ blood plasma between the different production stages (finishing phase and rearing phase).

The results found in PCA of the plasma metabolome in the rearing phase (Figure 1), finishing phase (Figure 2), and between these both phases (Figure 3) are similar. The distribution of all analyses data showed an overlap between all groups and it was not possible to observe a clustering among the treatments or between the different production stages. This may indicate that the metabolite profile presented only a few or no variables expressed differentially among treatments. In the rearing phase, the two principal components together explain 50.6% of the total variance (PC1 = 32.6%; PC2 = 18.0%). In the finishing phase, the two principal components together explain 51.4% of the total variance (PC1 = 31.3%; PC2 = 20.3%). Between the times (rearing and finishing phases), the two principal components together explain 46.1% of the total variance (PC1 = 30.3%; PC2 = 15.8%).

### 3.2. Supervised Analysis of Metabolome (Multiple Linear Regression)

Regarding the results found in the supervised analysis of the rearing phase (Table 2), six metabolites were identified as differentially expressed according to the prenatal treatment received. The NP group had higher levels of plasmatic carnosine than the groups that received protein-energy supplementation (PP and FP). The NP treatment also showed higher levels of putrescine than the PP group and higher levels of Trans-4-Hydroxy-L-Proline (t4-OH-Pro) and tryptophan than the FP treatment. Citrulline, on the other hand, showed higher levels in the FP treatment and showed a significant difference with the NP treatment. Finally, SM C18:1 showed a significant difference between FP and PP treatments, with the FP treatment having the highest concentrations.

In the finishing phase (Table 3), just three metabolites were differentially expressed among the treatments. C5:1-DC was the most significant metabolite among the treatment (*p* = 0.001), where the group FP showed higher levels compared to other treatments (NP and PP). The FP treatment also had higher levels of SM C26:0 compared to NP and PP groups. Lastly, the group PP showed higher levels of Serotonin in comparison to the group not supplemented (NP), and showed no differences with FP treatment.

The results of all expressed metabolites in both production stages can be better visualized in Table S1 (rearing phase) and Table S2 (finishing phase).

### 3.3. Repeated Measures Analysis over Time

Regarding the results in the repeated measures analysis over time, we found 21 significant metabolites for the variable “Time” and none for the interaction of “Treatment × Time” (all *p* values > 0.05). The significant results can be viewed in Table 4 and all the results regardless of the *p* value can be found in Table S3.

### 3.4. Functional Enrichment

**Figure 4:**
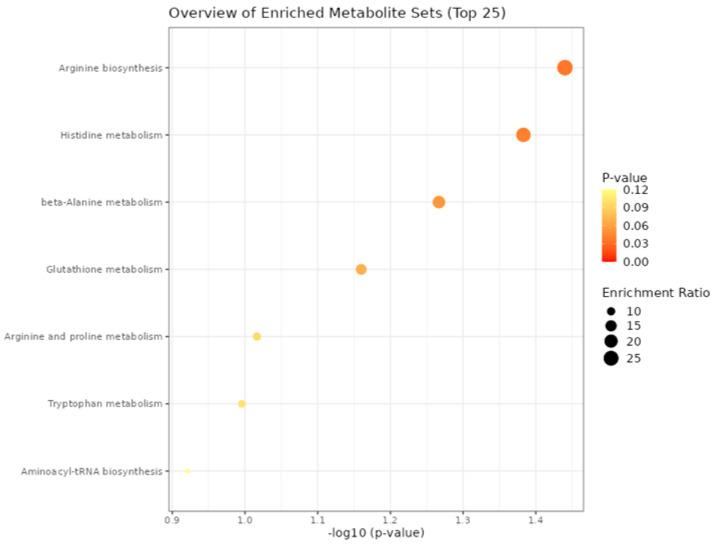
Top biological processes involved with significant blood plasma metabolites of bulls in the rearing phase from the three different maternal treatments (NP, PP, and FP).

In the enrichment analysis of plasma metabolites of bulls, we found the top biological processes related to differentially expressed metabolites among the prenatal treatments in the rearing phase (Figure 4) and in the finishing phase. In the finishing phase, as we found just three differential metabolites expressed, it was identified no significant biological processes related. However, in the rearing phase we found two significant processes related to the set of differentially expressed metabolites. Between them, Arginine biosynthesis (*p* = 0.036) and Histidine metabolism (*p* = 0.041) were considered the 2 enriched significant metabolic processes.

## 4. Discussion

According to our literature search, this is the first study that assessed the impact of three different prenatal supplementation approaches on the plasma metabolome of Nellore bulls in the rearing and finishing phase. This study is innovative and may contribute to the understanding of some molecular mechanisms involving maternal nutrition and long-term effects on offspring in beef cattle.

Based on our results, we selected the main classes and metabolites differentially expressed among the treatments to discuss briefly. In addition, we also discuss the metabolic pathways and the differences found between the production phases (rearing and finishing).

Carnosine is a dipeptide composed of beta-alanine and histidine. In cattle, this metabolite is found in greater amounts in skeletal muscle [27] and has several functions related to homeostasis and epigenetic regulation [28,29,30,31]. Furthermore, carnosine levels have already been positively correlated with feed efficiency in Nellore cattle [32]. Thus, observing only the levels of this metabolite, we could conclude that animals from treatments that received protein-energy supplementation during the prenatal period (PP and FP) may have lower feed efficiency than animals from the control treatment (NP) in the rearing phase. However, complex phenotypes, such as feed efficiency, are controlled by several genetic mechanisms, which makes it difficult to conclude based only on the concentration level of one metabolite.

Putrescine is a polyamine derived from Arginine, naturally found in all organisms [33]. This metabolite has some functions related to epigenetic mechanisms (DNA topology; [34]) and can improve the response of proteins to heat shock [35]. Additionally, according to Liao et al. [36], putrescine levels may also be related to adaptability to heat stress in cattle. Thus, the results of our study may be indicative that progenies from cows that did not receive protein-energy supplementation (NP) may have lower susceptibility to heat stress than the progenies of the PP group (supplementation in the final third of pregnancy) in the rearing phase.

T4-OH-Pro is a biogenic amine that is associated with type 2 diabetes mellitus [37] and with an increased risk of prostate cancer [38] in humans. In pregnant cattle, T4-OH-Pro in blood serum was associated with the group of cows with higher body condition scores in early lactation [39]. In addition, a correlation between the blood concentration of T4-OH-Pro and the level of lipolysis in early lactation in cows has also been reported [40]. In our study, the control treatment (NP) during the rearing phase showed the highest levels of this metabolite and differed from the treatment in which it received energy protein supplementation throughout pregnancy (FP). This may be indicative that progenies from FP cows may have lower rates of lipolysis than the NP group.

Citrulline is a non-essential amino acid considered a biomarker for enterocyte mass, epithelial cell damage, and absorptive function [41,42]. The administration of citrulline to pregnant ewes and cows improves embryo survival [43] and tends to increase the number of antral follicles in the ovaries of ewes [44]. Furthermore, citrulline has several functions: protein synthesis, intestinal homeostasis, nitrogen balance, growth and development, anti-oxidation, muscle performance, intestinal functions, renal function, exercise performance, blood pressure, vasodilation, and anti-inflammatory action [45,46,47,48]. Given these numerous functions, in the rearing phase, animals from the FP treatment (higher concentration of citrulline) may have advantages over the control treatment (NP). This is mainly related to growth and muscle performance, which are the central objectives of beef cattle production.

Tryptophan is an essential amino acid that plays a major role in protein synthesis [49]. This metabolite also has several other important functions and metabolic pathways related to serotonin synthesis [50,51], kynurenine synthesis [52,53], and synthesis of melatonin [49]. These biological pathways are related to immunological modulatory effects [54]. The higher level of tryptophan found in the NP treatment animals may indicate greater resilience to stressors compared to the FP treatment in the rearing phase.

Serotonin, or 5-Hydroxytryptamine, is a biogenic amine with several effects on the central nervous system [55], and functions in the regulation of energy metabolism, lactation, and calcium homeostasis [56,57]. The synthesis of serotonin occurs naturally using tryptophan in the diet [50]. Specifically, in animal production, the roles of serotonin are related to feed intake control [58], energy metabolism [59], stress [60], immunological system [61], mineral homeostasis [62], and hormone release [63]. Serotonin has been studied in cattle, but the metabolism and manipulation of this metabolite have received limited investigations [64].

C5:1-DC (Glutaconylcarnitine) is a metabolite belonging to the acylcarnitine class. Acylcarnitines are esters of fatty acids and L-carnitine [65]. Due to a large number of constituents and structures, the acylcarnitines play important roles in cell metabolism [66]. Among its specific functions have: regulating the balance of intracellular sugar and lipid metabolism [67], metabolism of branched-chain amino acids [68], homeostasis of the mitochondrial acyl-CoA/CoA ratio, regulation of glucagon/insulin [69], oxidation of fatty acids [70], and others. This metabolite class is related to several metabolic diseases [71], however, the relationship with animal husbandry is scarce in the literature. According to Ladeira et al. and Nguyen et al. [72,73], acylcarnitine is associated with the deposition of intramuscular fat in beef cattle, which may affect the marbling of the meat. In our study, the different levels of C5:1-DC found among FP treatment and the others (PP and NP) may be related mainly with marbling and with the metabolism of amino acids, as we identified in the significant biological processes in the rearing phase.

SM C18:1 and SM C26:0 are types of sphingolipids of the sphingomyelin class. Among the sphingolipids, this class of metabolites is the most abundant component of the cellular plasma membrane in mammals, preferentially associating with cholesterol to form stronger lipid bonds [74]. These bonds play a major role in signal transduction in many cell types and in maintaining membrane integrity [75,76,77]. Sphingomyelins are also correlated with parameters of obesity, insulin resistance, liver function, and lipid metabolism [78]. More studies are still needed in the area of prenatal nutrition and animal production in order to understand the maternal effects on the offspring’s metabolically levels of sphingomyelins. Additionally, the other classes of metabolites discussed here in this study need deeper information about their functions and roles in animal production and fetal programming. With this, it will be possible, in fact, to understand the molecular mechanisms that involve the phenotype.

Regarding the metabolome differences found between the production stages (rearing and finishing phases), we already expected plasmatic concentration levels to divergent in some metabolites. The different environments which the bulls were exposed (extensive production x intensive production) when comparing the period of the rearing phase and finishing phase were completely different in terms of nutrition and stressful factors. Due to the use of a greater proportion of grains and other products during the finishing phase, these foods can lead to digestive disorders in animals [79]. Other critical points are related to high farm animal density in the feedlot and the process of adaptation in a new environment [80], which may affect the productive indices of the herd and trigger stressful responses. All of these factors impact the metabolic state of the animals, showing differences in the plasma metabolome when we compare the rearing phase and finishing phase. Furthermore, we have not found an interaction between time and treatment in the analysis. Thus, the effect that we observed in the repeated measures analysis over time is just related to the difference caused by the production stages.

In the enrichment analysis, the significant biological processes affected by prenatal treatments in the rearing phase were related to Arginine biosynthesis and Histidine metabolism. No significant metabolic pathway was found in the finishing phase due to the lower number of differential metabolites expressed among the treatments (only 3 metabolites). The significant metabolic pathways identified are related to amino acid metabolism, therefore implying changes in protein metabolism. As the different maternal supplementation approaches (NP, PP, and FP) were based on protein and energy levels, this impacted on the protein metabolism in the rearing phase (Arginine biosynthesis and Histidine metabolism). According to Schalch Junior et al. [7], the top metabolic pathway affected by prenatal nutrition in calves (30 days old) was the Histidine metabolism, showing similar effects with the present study when compared to the rearing phase.

## 5. Conclusions

The different prenatal supplementation approaches (NP, PP, and FP) influenced the plasma metabolome of bulls in both the rearing phase and the finishing phase. In addition, we found biological pathways (amino acid metabolism) affected by prenatal nutrition in the rearing phase and metabolites differentially expressed over time (between the rearing phase and finishing phase). The most significant metabolites found in the study are related to protein metabolism, which could be expected due to the protein supply differences in the prenatal nutritional approaches. In summary, these findings corroborate the understanding of the part of the molecular mechanisms that involve fetal programming.

## Figures and Tables

**Table 1:** Ingredients and nutrient content of the dams’ supplement.

Ingredients	Mineral Supplement	Protein-Energy Supplement
Corn (%)	35	60
Soybean meal (%)	-	30
Dicalcium phosphate (%)	10	-
Urea 45% (%)	-	2.5
Salt (%)	30	5
Minerthal 160 MD (%) *	25	2.5
Total digestible nutrients (%)	26.76	67.55
Crude protein (%)	2.79	24.78
Non-protein nitrogen (%)	-	7.03
Acid detergent fiber (%)	1.25	4.76
Neutral detergent fiber (%)	4.29	11.24
Fat (%)	1.26	2.61
Calcium (g/kg)	74.11	6.2
Phosphorus (g/kg)	59.38	7.24

* Mineral premix composition (Minerthal company): Calcium = 8.6 g/kg; Cobalt = 6.4 mg/kg; Copper = 108 mg/kg; Sulfur = 2.4 g/kg; Fluorine = 64 mg/kg; Phosphorus = 6.4 g/kg; Iodine = 5.4 mg/kg; Manganese = 108 mg/kg; Selenium = 3.2 mg/kg; Zinc = 324 mg/kg; Sodium monensin = 160 mg/kg [10].

**Table 2:** Plasma significant metabolites (µM; mean ± standard error) of bulls in the rearing phase submitted to the different prenatal nutrition approaches (NP, PP, and FP) with their respective *p* values.

Metabolites	NP	PP	FP	*p* Value
Carnosine	23.69 ± 0.749 ^a^	18.68 ± 0.882 ^b^	19.44 ± 1.111 ^b^	0.008
Putrescine	0.128 ± 0.014 ^a^	0.076 ± 0.011 ^b^	0.090 ± 0.005 ^ab^	0.020
t4-OH-Pro	41.66 ± 2.435 ^a^	35.76 ± 1.985 ^ab^	33.76 ± 0.838 ^b^	0.032
Tryptophan	41.57 ± 3.867 ^a^	38.70 ± 2.632 ^ab^	31.26 ± 1.178 ^b^	0.037
Citrulline	48.51 ± 2.712 ^a^	52.72 ± 3.223 ^ab^	59.54 ± 1.657 ^b^	0.041
SM C18:1	6.805 ± 2.072 ^ab^	1.817 ± 0.997 ^a^	9.108 ± 2.147 ^b^	0.047

The small letters overwritten represent the significant contrasts. NP—not programmed; PP—partial programming; FP—fully programmed.

**Table 3:** Plasma significant metabolites (µM; mean ± standard error) of bulls in the rearing phase submitted to the different prenatal nutrition approaches (NP, PP, and FP) with their respective *p* values.

Metabolites	NP	PP	FP	*p* Value
C5:1-DC	0.008 ± 0.006 ^a^	0.005 ± 0.005 ^a^	0.012 ± 0.009 ^b^	0.001
SM C26:0	0.068 ± 0.032 ^a^	0.049 ± 0.006 ^a^	0.384 ± 0.090 ^b^	0.002
Serotonin	0.414 ± 0.047 ^a^	0.910 ± 0.176 ^b^	0.646 ± 0.080 ^ab^	0.026

The small letters overwritten represent the significant contrasts. NP—not programmed; PP—partial programming; FP—fully programmed.

**Table 4:** Repeated measures analysis over time with the significant metabolites at least in one *p* value.

Metabolites	*p* Values	
	Time	Interaction (Treatment × Time)
Taurine	<0.001	0.593
Carnosine	<0.001	0.933
Histidine	<0.001	0.825
Proline	<0.001	0.626
Sarcosine	<0.001	0.969
Glutamine	<0.001	0.406
Tryptophan	<0.001	0.544
Serine	<0.001	0.825
Leucine	<0.001	0.368
Ornithine	0.001	0.145
Asparagine	0.001	0.968
Met-SO	0.001	0.175
Arginine	0.001	0.459
Creatinine	0.002	0.184
Lysine	0.002	0.474
Methionine	0.004	0.781
ADMA	0.009	0.963
Glycine	0.009	0.075
Kynurenine	0.015	0.268
Citrulline	0.023	0.071
C9	0.026	0.820

## Data Availability

The data presented in this study are available on request from the corresponding author. The data are not publicly available due to privacy.

## Supplementary Materials

The following supporting information can be downloaded at: https://www.mdpi.com/article/10.3390/metabo13020259/s1, Table S1: Plasma metabolites (µM; mean ± standard error) of bulls in the rearing phase submitted to the different prenatal nutrition approaches (NP, PP and FP) with their respective *p* values. Table S2: Plasma metabolites (µM; mean ± standard error) of bulls in the rearing phase submitted to the different prenatal nutrition approaches (NP, PP and FP) with their respective *p* values. Table S3: Repeated measures analysis over time with all metabolites and the respective *p* values.
